# National variation in acute kidney injury diagnosis and monitoring across adult intensive care units in china: a nationwide cross-sectional survey

**DOI:** 10.1016/j.aicoj.2026.100166

**Published:** 2026-09-22

**Authors:** Xinran Wang, Xiuhua Zhang, Weiting Zhang, Yu Xin, Pengfei Huang, Yanqi Liu, Kai Zeng, Xianglin Meng, Zhiyong Peng, Wenxiong Li, Zhenjie Hu, Yingzi Huang, Meili Duan, Mingyan Zhao, Kaijiang Yu, Changsong Wang

**Affiliations:** a Department of Critical Care Medicine, The First Affiliated Hospital of Harbin Medical University, No. 23 You Zheng Street, Harbin 150001, Heilongjiang Province, China; b Heilongjiang Provincial Key Laboratory of Critical Care Medicine, Harbin 150001, China; c Department of Critical Care Medicine, Zhongnan Hospital of Wuhan University, No. 169 East Lake Road, Wuchang District, Wuhan 430071, Hubei Province, China; d Department of Critical Care Medicine, Beijing Chao-Yang Hospital, Capital Medical University, No. 8 Gongren Tiyuchang Nanlu, Chaoyang District, Beijing 100020, China; e Department of Critical Care Medicine, The Fourth Hospital of Hebei Medical University, No. 12 Jiankang Road, Shijiazhuang 050011, Hebei Province, China; f Jiangsu Provincial Key Laboratory of Critical Care Medicine, Department of Critical Care Medicine, Zhongda Hospital, School of Medicine, Southeast University, No. 87 Dingjiaqiao Road, Gulou District, Nanjing 210009, Jiangsu Province, China; g Department of Critical Care Medicine, Beijing Friendship Hospital, Capital Medical University, No. 95 Yongan Road, Xicheng District, Beijing 100050, China

**Keywords:** Acute kidney injury, Intensive care units, Urine output monitoring, serum creatinine, Critical care, Quality improvement, China

## Abstract

**Background:**

Acute kidney injury (AKI) is common in critically ill patients and strongly associated with adverse outcomes, yet national ICU-level evidence on AKI diagnosis and monitoring in China remains limited.

**Methods:**

We conducted a nationwide anonymous cross-sectional web survey of adult ICUs in China from June 25 to September 26, 2025. The survey assessed AKI diagnosis and staging, UO and SCr monitoring across clinical scenarios, biomarker use, early warning systems, and changes in UO monitoring after AKI. We used multivariable logistic regression to identify factors associated with UO-monitoring practices, with FDR correction for multiple comparisons. An exploratory 0–100-point AKI-MIS summarised ICU-level monitoring implementation, with higher scores indicating broader implementation.

**Results:**

Of 2,002 responses, 1,896 unique adult ICU units were included. AKI diagnosis was reported by 97.9% of ICUs, whereas 63.2% explicitly implemented staging and 75.9% used both UO and SCr for diagnosis. Hourly UO monitoring increased from 49.6% in routine practice to 74.1% in sepsis, 75.0% in AKI, and 77.7% in sepsis-associated AKI. Dynamic UO monitoring was implemented in 14.8% of ICUs; 83.5% reported increased nursing workload, and 69.9% identified workload or insufficient staffing as the main barrier. After FDR correction, geographic region, nurse-to-bed ratio, and baseline monitoring intensity were associated with UO-monitoring practices. The median AKI-MIS was 68.1 (IQR, 60.3–74.9), with higher adjusted scores in teaching hospitals.

**Conclusions:**

Among participating adult ICU units across all 31 provincial-level administrative regions of mainland China, AKI diagnosis and monitoring were widely reported, but substantial heterogeneity remained across monitoring systems. These findings provide a broad descriptive benchmark and identify priorities for strengthening standardisation, risk-responsive escalation, and closed-loop response pathways.

## Background

Acute kidney injury (AKI) is a common and clinically important syndrome in adult intensive care units (ICUs), associated with substantial short- and long-term morbidity, mortality, and healthcare costs [[1], [2], [3]]. The 2026 KDIGO AKI/AKD public review draft continues to recommend using changes in both serum creatinine (SCr) and urine output (UO) for AKI diagnosis and staging, supporting a dual-criterion framework for recognition and risk stratification [4]. In the multinational AKI-EPI study, AKI occurred in more than half of ICU patients, and mortality increased progressively with higher KDIGO stage, underscoring both the high burden of AKI in critical illness and its close association with adverse outcomes.^1^ More recent prospective multinational evidence from the EPIS-AKI study, which enrolled 10,568 major surgical patients requiring postoperative ICU or high-dependency care across 30 countries, found postoperative AKI in 18.4%, with progressively worse outcomes at higher KDIGO stages [5].

UO monitoring is a key component of this diagnostic and staging framework because oliguria may precede rises in SCr, may also occur without concomitant creatinine changes, and provides prognostic information beyond SCr-based staging [[6], [7], [8], [9]]. Prospective and observational ICU studies have shown that oliguria is not only an early warning sign of AKI but is also independently associated with adverse outcomes, including mortality [[6], [7], [8]]. In addition, the severity and duration of oliguria are closely related to AKI diagnosis, AKI severity classification, and prognosis [7,8]. Higher-frequency or real-time UO monitoring may facilitate earlier recognition of AKI [10,11]. More intensive UO monitoring has also been associated with better outcomes in observational studies [10,11]. Studies have further shown that inclusion or exclusion of UO criteria can materially alter epidemiologic estimates and prognostic stratification of AKI, suggesting that reliance on SCr criteria alone may underestimate AKI burden and lead to risk misclassification.^9^,^12^] Multicenter work on standardizing large UO datasets has also shown that consistent application of UO criteria across centers poses methodological challenges, further highlighting the complexity of implementing UO monitoring at scale [13]. Compared with laboratory-driven SCr monitoring, UO monitoring is more susceptible to variation in charting, data processing, and clinical interpretation [11,13].

Beyond bedside measurement itself, the quality of AKI monitoring is strongly influenced by systems and resource factors, including staffing, workflow standardisation, digital infrastructure, and escalation pathways. These factors jointly determine whether oliguria is detected in a timely manner and translated into effective clinical decision-making [[14], [15], [16]]. With respect to implementation strategies, prior studies have shown inconsistent effects across approaches: in high-risk cardiac surgery patients, biomarker-guided implementation of a KDIGO care bundle reduced the incidence and severity of AKI [17], whereas electronic alert trials have shown less consistent benefit on patient outcomes [15,16], although some studies suggest improvement in selected care processes [16]. Benchmark data on contemporary AKI diagnosis and monitoring practices are therefore important for identifying actionable gaps and informing scalable quality-improvement and health-system strategies.

In China, AKI likewise represents an important public health issue, and prior studies have repeatedly highlighted high rates of missed diagnosis and under-recognition. A nationwide cross-sectional survey reported a substantial burden of AKI among hospitalized patients together with a high non-recognition rate, indicating considerable room for improvement in detection and care pathways [18]. Meanwhile, national authorities and professional societies have increasingly emphasised strengthening ICU capacity, staffing, equipment allocation, and quality management, further underscoring the need for up-to-date ICU unit-level evidence to characterise the real-world status of AKI diagnosis and monitoring [14]. However, nationwide ICU-unit–level evidence remains limited on how adult ICUs in China operationalise guideline-concordant AKI diagnosis, including the combined use of SCr and UO, the intensity of UO and SCr monitoring under routine and higher-risk conditions, and the implementation of manual versus automated workflows, alert thresholds, and closed-loop response pathways.

Accordingly, we conducted a nationwide anonymous cross-sectional survey, under the auspices of the Nephrology Group of the Chinese Society of Critical Care Medicine and the Nephrology Quality Control Group of China National Critical Care Quality Control Centre, to characterise national variation in AKI diagnosis and monitoring across adult ICUs in China. The survey focused on key domains including diagnostic basis, UO and SCr monitoring, biomarker use, and early warning systems. Our objectives were to characterise regional and resource-related variation in ICU AKI monitoring implementation and to identify system-level factors associated with intensified monitoring. The study was designed and reported in accordance with relevant guidance for survey and observational research [19,20].

## Methods

### Study design and governance

This was a nationwide, anonymous, cross-sectional web-based survey designed to characterise national variation in AKI diagnosis and monitoring across adult ICU units in China. The survey was jointly initiated and organised by the Nephrology Group of the Chinese Society of Critical Care Medicine and the Nephrology Quality Control Group of China National Critical Care Quality Control Centre. The questionnaire focused on core domains including the basis for AKI diagnosis, UO and SCr monitoring, biomarker use, and early warning/alert workflows. AKI-related concepts were defined in accordance with KDIGO guidance [4,21]. The Ethics Committee of First Affiliated Hospital of Harbin Medical University determined that this anonymous questionnaire survey of ICU units, which collected no patient information, was exempt from ethics review. Completion of the questionnaire was taken as voluntary participation.

### Survey development and reporting standards

Questionnaire items were developed through expert consensus, iterative revision, and internal review to ensure clinical relevance and clarity. Because this was an internet-based survey, its conduct and reporting were informed, where applicable, by CHERRIES recommendations, including completeness requirements for key items, handling of potential duplicate submissions, and transparent reporting of item-level missingness [22]. The study was also reported in accordance with STROBE and general principles for survey research [20,23].

### Survey administration

The survey was administered online using Wenjuanxing (Questionnaire Star), a web-based survey platform, and data were collected between June 25 and September 26, 2025. Similar online questionnaire-based approaches have previously been used in population-based survey research in China [24]. The survey link and QR code were disseminated through professional academic networks and collaborating ICU communities to maximize nationwide coverage, specifically through WeChat groups involving members of the Nephrology Group of the Chinese Society of Critical Care Medicine, the Nephrology Quality Control Group of China National Critical Care Quality Control Centre, and provincial collaborating networks. Similar nationwide ICU survey models based on web-based, anonymous, and QR code-enabled participation have also been implemented in China [25]. The survey invitation requested that the questionnaire be completed by the ICU director or head nurse, with one response submitted per adult ICU unit; however, respondent role was not collected or independently verified. Three reminders were posted in the same WeChat groups during the survey period. Participation was voluntary, and no hospital identifiers were collected. Platform settings required responses to key routing and core outcome items. Item-level missingness was summarised and reported separately. Because the survey was distributed through open professional groups rather than to a predefined list of eligible ICUs, the number of invited ICUs and the response rate could not be determined.

### Data quality checks and deduplication

Prespecified data-quality checks were implemented to identify logical inconsistencies and implausible response combinations, and potential duplicate submissions. Records with clearly correctable logical conflicts were reconciled after manual review, whereas ambiguous responses were left unchanged. To minimize duplicate submissions from the same ICU unit, potential duplicates were screened using two composite identifiers based on reported geographic location, hospital level and type, teaching status, ICU type, and ICU-bed number; the second additionally included physician and nurse numbers. Submission timing, IP address, IP-derived geographic location, and response-pattern similarity were used as supporting information during manual adjudication. When multiple submissions were judged to represent the same ICU unit, the most complete and internally consistent record was retained, without merging item-level responses. Records were retained separately when duplication could not be established with sufficient confidence. Platform metadata were used only for data-quality assessment and were not included in the analytical dataset. Because no hospital identifiers were collected and ICU units served as both the survey unit and the unit of analysis, coverage was described by the number and geographic distribution of responding ICU units.

### Outcomes and covariates

The primary outcome was whether an ICU unit performed hourly UO monitoring under routine practice (Model A), defined as a routine UO monitoring frequency of once per hour. Two AKI-specific outcomes were also evaluated: hourly UO monitoring during AKI among all ICU units (Model B) and escalation of UO-monitoring frequency during AKI among units without routine hourly monitoring (Model C). Model B additionally included routine UO-monitoring frequency as a covariate. Escalation was defined as a shorter scheduled monitoring interval during AKI than under routine practice; a transition from irregular routine monitoring to any scheduled interval was also considered escalation. A full-sample analysis of monitoring escalation was conducted as a sensitivity analysis (Model D). Candidate determinants included region, hospital level, teaching status, hospital type, ICU type, and ICU resource indicators, including ICU-bed category, annual ICU-admission category, and nurse-to-bed-ratio category. Physician and nurse headcounts were described but were not included in the multivariable models because of their strong correlations with ICU bed numbers and with each other. To reduce the influence of extreme values and facilitate interpretation, resource-related variables were grouped according to the sample distribution and interpretable thresholds. Nurse-to-bed ratio was categorised as <2.5, 2.5 to <3.0, and ≥3.0 nurses per bed, with categories defined with reference to the recommended ICU nursing staffing range in current Chinese critical care guidelines [14].

### AKI Monitoring Implementation Score (AKI-MIS)

To summarise ICU-level implementation of AKI monitoring, we constructed an investigator-developed exploratory AKI Monitoring Implementation Score (AKI-MIS) from prespecified questionnaire items. The score comprised four domains: (1) detection and surveillance structure, including AKI diagnosis, staging, combined use of UO and SCr, early warning, and eGFR calculation; (2) monitoring intensity and escalation, covering UO and SCr monitoring across routine and high-risk scenarios; (3) standardisation and guideline concordance, including severity-based frequency adjustment, UO documentation units, and oliguria thresholds; and (4) digitalisation and response loop, including dynamic monitoring, clinician notification, EMR integration, and physician response. Routine-to-AKI escalation was also analysed separately. AKI-MIS was conceived as a formative composite measure, in which individual items jointly define different aspects of implementation rather than reflect a single latent construct [26,27].

The raw score ranges for Domains 1–4 were 0–10, 0–18, 0–10, and 0–11, respectively. To assign equal weight to each domain, raw domain scores were linearly transformed to a 0–25 scale. The total AKI-MIS was calculated as the sum of the four transformed domain scores (range, 0–100). Equal domain weighting prevented domains containing more items from dominating the total and did not imply equal clinical importance [27,28]. Higher scores indicated broader implementation of the included monitoring processes, not better patient outcomes or overall care quality. AKI-MIS was analysed only as a continuous exploratory measure without categorical cut-offs. Detailed scoring rules are provided in Supplementary Table S7, and an alternative weighting analysis is reported in Supplementary Table S8.

## Statistical analysis

Categorical variables were summarised as counts and percentages, and continuous variables as medians with IQRs. Denominators varied according to the number of valid responses for each item. Descriptive analyses used available responses, whereas multivariable analyses used complete cases; no missing values were imputed, and item-level missingness is reported in Supplementary Table S4. Multivariable logistic regression models were fitted for Models A–D, and adjusted odds ratios (aORs) with 95% CIs were reported; Model D is reported in Supplementary Table S5. Correlations among resource variables were assessed using Spearman’s coefficients, and variance-inflation factors were examined for retained predictors. Physician and nurse headcounts were excluded from the final models because of high collinearity with ICU bed numbers and with each other; the correlation matrix is provided in Supplementary Table S6. Benjamini–Hochberg false-discovery-rate (FDR)-adjusted q values were calculated separately across the non-reference contrasts within each multivariable model. Reported biomarker combinations were summarised descriptively. Regional differences in individual biomarker use were assessed using Pearson’s chi-square tests, with Monte Carlo P values for sparse tables and FDR correction across the seven biomarker-specific tests (Supplementary Table S9). For AKI-MIS, descriptive statistics and histograms were used to summarise its distribution. Associations between hospital and ICU characteristics and continuous AKI-MIS were estimated using multivariable ordinary least-squares regression with HC3 robust standard errors. Adjusted mean differences with 95% CIs were reported, with FDR correction across the non-reference contrasts; the alternative weighting analysis used the same model specification (Supplementary Table S8). Hospital-level clustering could not be modelled because hospital identifiers were not collected. Analyses were done in R (version 4.4.2). All tests were two-sided, and FDR-adjusted q values less than 0.05 were considered statistically significant.

## Results

During the survey period, 2,002 responses were received. No questionnaire was excluded for incompleteness because key items were mandatory. Ten records with clear, resolvable branch-logic inconsistencies were reviewed and recoded according to the data-quality rules described above. Manual adjudication identified and removed 106 duplicate submissions, leaving 1,896 unique adult ICU units from all 31 provincial-level administrative regions for analysis (Fig. 1 and Supplementary Figure S1). For non-mandatory resource variables, analyses used available observations; missingness was minimal (0–7 records per resource variable) and is detailed in Supplementary Table S4. Supplementary Table S1 provides a descriptive province-level comparison with published counts of hospitals with comprehensive ICUs.

**Fig. 1:**
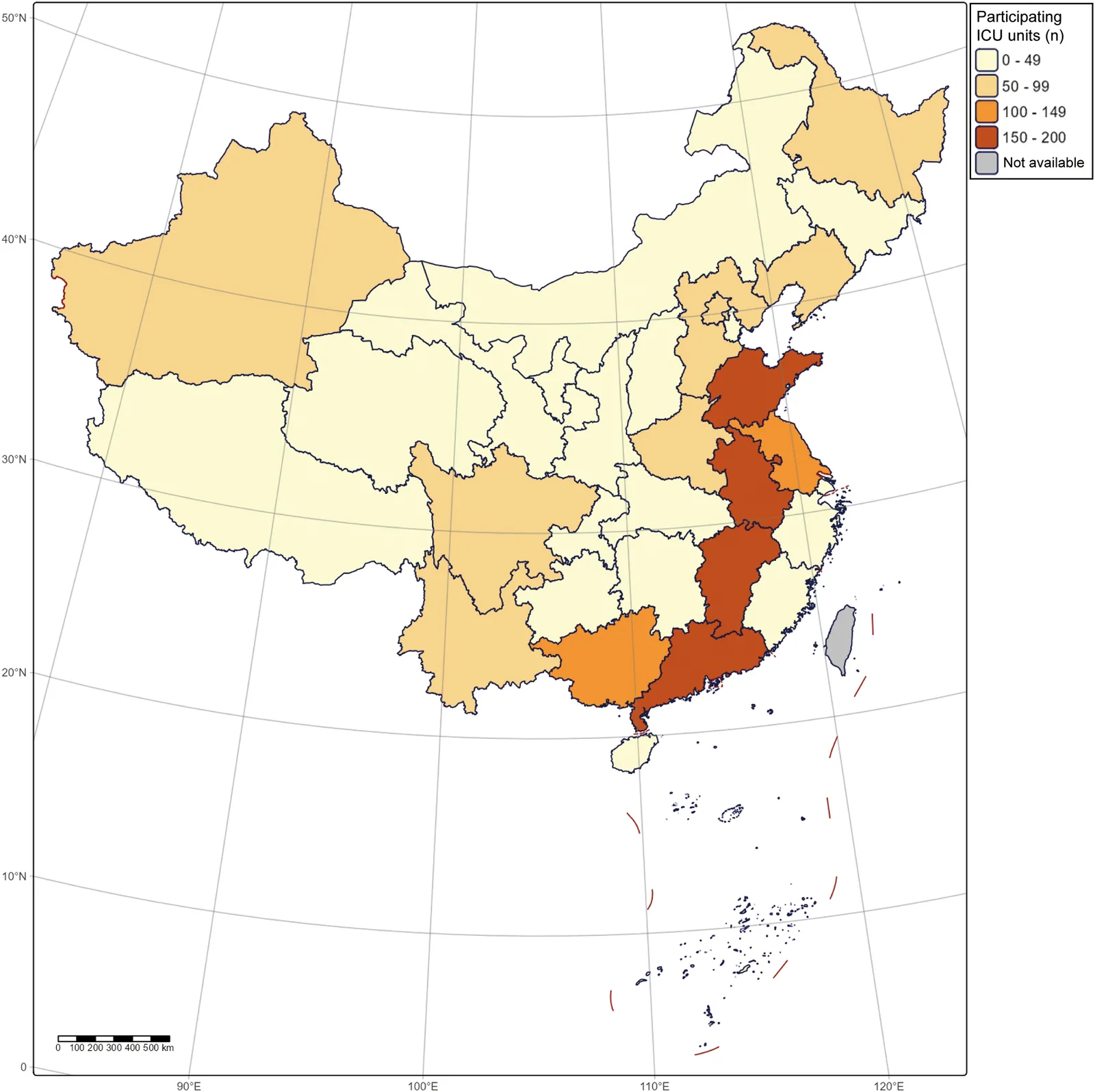
Geographic distribution of participating adult ICU units across mainland China. Province-level choropleth map showing the geographic distribution of the 1,896 participating adult ICU units across mainland China. Colour shading represents the number of participating ICU units in each provincial-level administrative region. ICU, intensive care unit.

The final analytic sample included 1,896 adult ICU units spanning all 31 provincial-level administrative regions and all seven major geographic regions of mainland China (Table 1 and Supplementary Table S1). East China contributed 655 units (34.5%), followed by South China (283, 14.9%), North China (242, 12.8%), Southwest China (241, 12.7%), Northeast China (198, 10.4%), Northwest China (174, 9.2%), and Central China (103, 5.4%). Supplementary Table S1 presents a descriptive province-level comparison between the participating ICU units and published numbers of hospitals with comprehensive ICUs. The survey and all analyses were conducted at the ICU-unit level.

**Table 1:** Geographic distribution of participating adult ICU units and contextual comparison with previous national critical care surveys.

Region	Current survey: adult ICU units, 2025 (N = 1,896)	National ICU survey: hospitals, 2022 (N = 2,005)	National critical care resource survey: hospitals with comprehensive ICUs, 2021–2023 (N = 4,744)
North China	242 (12.8%)	273 (13.6%)	538 (11.3%)
Northeast China	198 (10.4%)	208 (10.4%)	243 (5.1%)
East China	655 (34.5%)	494 (24.6%)	1,413 (29.8%)
Central China	103 (5.4%)	251 (12.5%)	670 (14.1%)
South China	283 (14.9%)	225 (11.2%)	621 (13.1%)
Southwest China	241 (12.7%)	374 (18.7%)	830 (17.5%)
Northwest China	174 (9.2%)	180 (9.0%)	429 (9.0%)
Total	1,896 (100.0%)	2,005 (100.0%)	4,744 (100.0%)

Data are n (%). Current study values are participating adult ICU units, whereas external survey values are hospitals. The external surveys are shown for descriptive comparison of regional composition. Regional counts for the national critical care resource survey were aggregated from published province-level hospital counts using the same seven-region classification. ICU, intensive care unit.

Participating ICU units were predominantly from tertiary hospitals (74.8%), teaching hospitals (67.0%), and general hospitals (93.8%), and most were general ICUs (90.1%) rather than specialised ICUs (Table 2). ICU capacity and staffing varied substantially across units. The median number of ICU beds was 15 (IQR, 10–23), with 28.0% of units having ≤10 beds, 23.0% having 11–15 beds, 24.3% having 16–23 beds, and 24.7% having ≥24 beds. Median staffing was 8 physicians (IQR, 6–12) and 26 nurses (IQR, 16–42). Among units with available data, 35.3% had ≤6 physicians, 19.9% had 7–8, 21.1% had 9–12, and 23.7% had ≥13; corresponding proportions for ICU nurses were 25.4%, 24.6%, 25.9%, and 24.1% for ≤16, 17–25, 26–42, and ≥43, respectively. Annual ICU admissions in the previous year had a median of 568 (IQR, 330–956), with 25.1% of units reporting ≤330 admissions, 25.1% reporting 331–568, 24.7% reporting 569–956, and 25.0% reporting ≥957. The median nurse-to-bed ratio was 1.77 (IQR, 1.42–2.19); among 1,894 units with available data, 85.5% had a ratio <2.5, 10.1% had a ratio of 2.5 to <3.0, and 4.4% had a ratio of ≥3.0.

**Table 2:** Characteristics of participating adult ICU units.

Characteristic	Level	Overall (N = 1,896)
Region (7 major regions)	East China	655/1,896 (34.5%)
	North China	242/1,896 (12.8%)
	Northeast China	198/1,896 (10.4%)
	Central China	103/1,896 (5.4%)
	South China	283/1,896 (14.9%)
	Southwest China	241/1,896 (12.7%)
	Northwest China	174/1,896 (9.2%)
Hospital level	Tertiary (including special grade)	1,419/1,896 (74.8%)
	Secondary	472/1,896 (24.9%)
	Other/unclassified	5/1,896 (0.3%)
Teaching hospital	Yes	1,270/1,896 (67.0%)
	No	626/1,896 (33.0%)
Hospital type	General	1,778/1,896 (93.8%)
	Specialty	118/1,896 (6.2%)
ICU type	General ICU	1,709/1,896 (90.1%)
	Specialised ICU	187/1,896 (9.9%)
ICU beds (number of beds)	Median (IQR)	15 (10–23)
	≤10	531/1,895 (28.0%)
	11–15	436/1,895 (23.0%)
	16–23	460/1,895 (24.3%)
	≥24	468/1,895 (24.7%)
	Missing	1/1,896 (0.1%)
ICU physicians (number of physicians)	Median (IQR)	8 (6–12)
	Missing	3/1,896 (0.2%)
ICU nurses (number of nurses)	Median (IQR)	26 (16–42)
	Missing	2/1,896 (0.1%)
Annual ICU admissions (past year)	Median (IQR)	568 (330–956)
	≤330	475/1,889 (25.1%)
	331–568	475/1,889 (25.1%)
	569–956	467/1,889 (24.7%)
	≥957	472/1,889 (25.0%)
	Missing	7/1,896 (0.4%)
Nurse-to-bed ratio (nurses per ICU bed)	Median (IQR)	1.77 (1.42–2.19)
	<2.5	1,619/1,894 (85.5%)
	2.5 to <3.0	191/1,894 (10.1%)
	≥3.0	84/1,894 (4.4%)
	Missing	2/1,896 (0.1%)

Data are n/N (%) unless otherwise indicated. Percentages for categorical variables were calculated using non-missing observations, whereas missingness was calculated using the full sample (N = 1,896). Continuous resource variables are summarized as median (IQR). ICU beds, annual ICU admissions, and nurse-to-bed ratio were categorized using the same cut points as in the final multivariable models. Physician and nurse headcounts are presented descriptively and were excluded from the primary models because of collinearity. Hospital level was classified with precedence: special-grade and tertiary hospitals were classified as tertiary; if both tertiary and secondary levels were selected, the ICU unit was classified as tertiary. Responses that could not be classified as tertiary or secondary after applying this rule were labelled as other/unclassified. Nurse-to-bed ratio was calculated as the number of ICU nurses divided by the number of ICU beds. ICU, intensive care unit; IQR, interquartile range.

AKI diagnosis was nearly universal among participating adult ICU units: 1,856 of 1,896 units (97.9%) reported diagnosing AKI in routine practice (Table 3). By contrast, only 1,198 units (63.2%) reported explicit routine implementation of AKI staging. Most ICUs used both urine output and serum creatinine in diagnosis, with 1,439 units (75.9%) indicating that the two criteria contributed approximately equally. In comparison, 407 units (21.5%) relied mainly on serum creatinine and only 50 units (2.6%) mainly on urine output. AKI recognition was widespread across adult ICUs in China, whereas explicit AKI staging and the relative use of functional diagnostic indicators varied across units.

**Table 3:** Core indicators of AKI diagnosis and monitoring in participating adult ICU units.

Practice indicator	Overall, n/N (%)
**AKI diagnosis**	
AKI diagnosis performed in the ICU	1856/1896 (97.9)
AKI staging explicitly implemented	1198/1896 (63.2)
Diagnostic basis: UO and SCr contributed approximately equally	1439/1896 (75.9)
**Urine output monitoring and implementation**	
Routine hourly UO monitoring	940/1896 (49.6)
Monitoring frequency adjusted according to illness severity	1589/1896 (83.8)
Dynamic urine output monitoring implemented	280/1896 (14.8)
Among units using dynamic monitoring: decreased UO triggers a physician alert	269/280 (96.1)
Among units using dynamic monitoring: data uploaded to the EMR	154/280 (55.0)
**Serum creatinine monitoring**	
Routine SCr monitoring at least daily	1340/1896 (70.7)
In AKI: SCr monitoring at least daily	1750/1896 (92.3)
In SA-AKI: SCr monitoring at least daily	1740/1896 (91.8)
Monitoring frequency adjusted according to illness severity	1679/1896 (88.6)
**Digital and advanced monitoring**	
AKI high-risk early warning system available	493/1896 (26.0)
eGFR routinely calculated	1267/1896 (66.8)
Cystatin C used	1334/1896 (70.4)
NGAL used	330/1896 (17.4)

Data are n/N (%). Percentages were recalculated from the final analytic dataset of 1,896 unique ICU units. At-least-daily serum creatinine monitoring included intervals of <12 h, every 12 h, and once daily. Physician-alert and electronic-medical-record-upload indicators used the 280 units reporting dynamic monitoring as the denominator. Complete scenario-specific urine output monitoring distributions are shown in Fig. 2. Detailed urine output measurement units and oliguria alert thresholds are presented in Supplementary Table S2; implementation perceptions and barriers are presented in Supplementary Table S3. Biomarker combinations and regional variation are presented in Supplementary Table S9.

Abbreviations: AKI, acute kidney injury; eGFR, estimated glomerular filtration rate; EMR, electronic medical record; ICU, intensive care unit; NGAL, neutrophil gelatinase-associated lipocalin; SA-AKI, sepsis-associated acute kidney injury; SCr, serum creatinine; UO, urine output.

Urine output monitoring intensified across higher-risk clinical scenarios (Fig. 2 and Table 3). Hourly urine output monitoring increased from 49.6% in routine practice to 74.1% in sepsis, 75.0% in AKI, and 77.7% in sepsis-associated AKI. Among the 940 units reporting routine hourly monitoring, 915 (97.3%) continued hourly monitoring during AKI. Among the 956 units without routine hourly monitoring, 733 (76.7%) moved to a shorter scheduled interval or from irregular to scheduled monitoring during AKI (Fig. 2B).

**Fig. 2:**
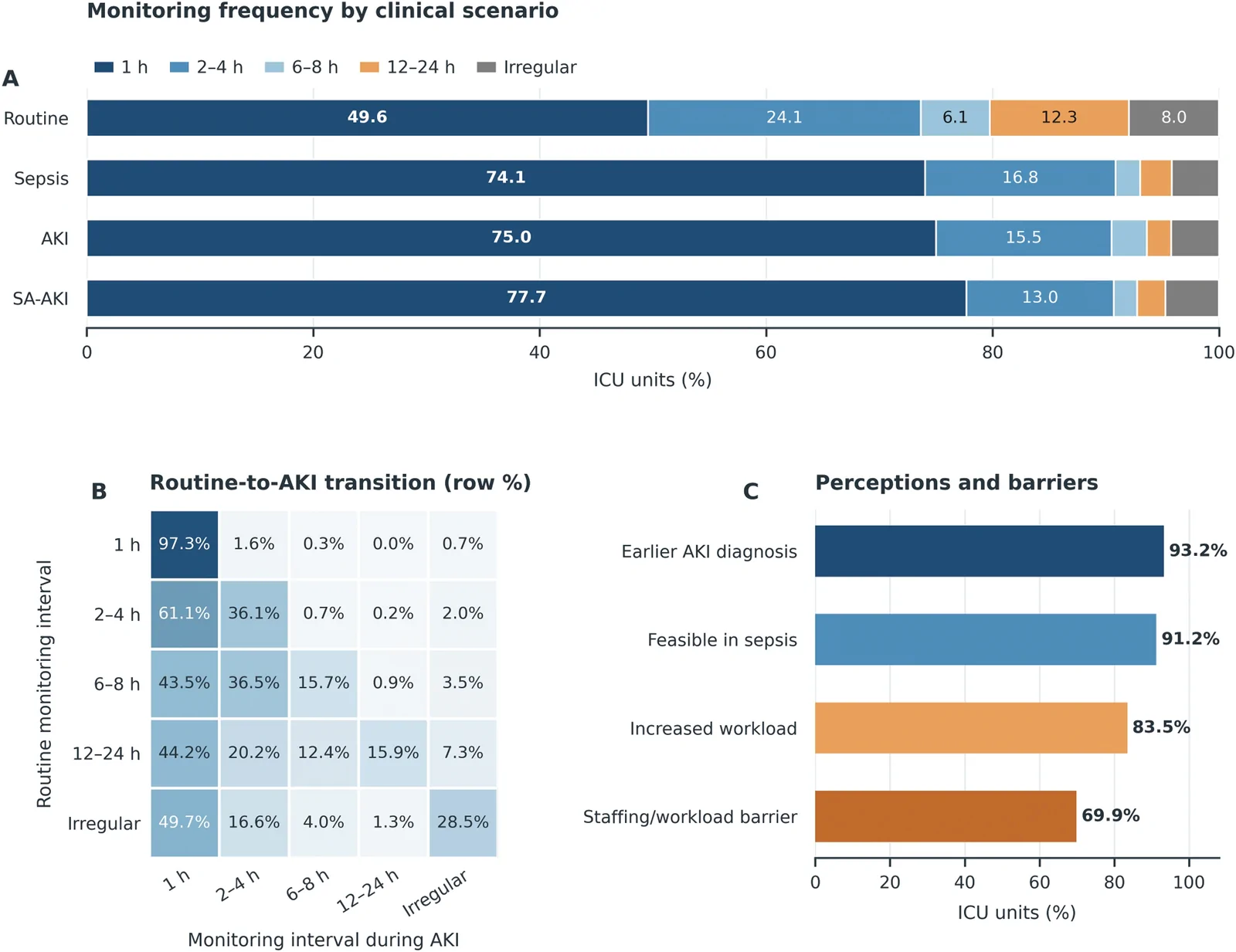
Urine-output monitoring frequency, routine-to-AKI transitions, and perceptions and barriers among 1,896 adult ICUs. (A) Distribution of urine-output monitoring intervals under routine conditions and in sepsis, AKI, and SA-AKI. For display, the original intervals were grouped as 1 h, 2–4 h, 6–8 h, 12–24 h, and irregular; values ≥5% are shown. (B) Row-normalized transition matrix from routine to AKI monitoring; cells show percentages within each routine-monitoring category. (C) Perceptions and barriers regarding intensified monitoring, including its perceived benefit for earlier AKI diagnosis, the feasibility of hourly monitoring in sepsis, increased workload, and staffing/workload constraints. AKI, acute kidney injury; ICU, intensive care unit; SA-AKI, sepsis-associated acute kidney injury.

Dynamic UO monitoring was reported by 280 of 1,896 units (14.8%), whereas 1,616 (85.2%) relied on manual measurement (Table 3). mL/h was the most common recording unit for both dynamic and manual monitoring (76.8% and 78.2%, respectively); 17.7% of manually monitored units recorded absolute volume in mL. Oliguria thresholds were recorded in weight-based units by 131 units, in mL/h by 1,479, and as absolute volume in mL by 286 (Supplementary Table S2). Among dynamic-monitoring units, 96.1% reported physician alerts for oliguria and 55.0% reported upload to the electronic medical record. Most units considered more frequent UO monitoring helpful for earlier AKI diagnosis (93.2%) and hourly monitoring feasible in sepsis (91.2%); 83.5% reported increased nursing workload, and 69.9% identified workload or insufficient staffing as the main barrier (Fig. 2C and Supplementary Table S3).

SCr monitoring also intensified in higher-risk settings. At-least-daily monitoring increased from 70.7% in routine practice to 92.3% in AKI and 91.8% in sepsis-associated AKI, and 88.6% of units adjusted monitoring frequency according to illness severity. An AKI high-risk early-warning system was available in 26.0% of units, and 66.8% routinely calculated eGFR.

Among named kidney-injury biomarkers, cystatin C was reported by 1,334 units (70.4%), neutrophil gelatinase-associated lipocalin by 330 (17.4%), interleukin-18 by 281 (14.8%), kidney injury molecule-1 by 143 (7.5%), the product of tissue inhibitor of metalloproteinases-2 and insulin-like growth factor-binding protein 7 by 72 (3.8%), and liver-type fatty acid-binding protein by 42 (2.2%). Within the six prespecified named biomarker fields, cystatin C as the only selected named biomarker was the most common pattern (50.3%), followed by neutrophil gelatinase-associated lipocalin plus cystatin C (6.8%) and interleukin-18 plus cystatin C (5.9%). After FDR correction, regional variation remained significant for neutrophil gelatinase-associated lipocalin use (q < 0.001) and selection of “Other” biomarkers (q = 0.008) (Supplementary Table S9).

In Model A (complete-case N = 1,888), South China versus East China (aOR, 2.13; 95% CI, 1.59–2.87; q < 0.001) and a nurse-to-bed ratio of 2.5 to <3.0 versus <2.5 (aOR, 1.61; 95% CI, 1.17–2.22; q = 0.034) were independently associated with routine hourly UO monitoring (Table 4 and Fig. 3A). In Model B (N = 1,888), routine monitoring every 2–4 h (aOR, 1.85; 95% CI, 1.42–2.41; q < 0.001) and every hour (aOR, 42.06; 95% CI, 27.10–65.30; q < 0.001), compared with every ≥6 h or irregular monitoring, were associated with hourly monitoring during AKI (Fig. 3B). In Model C, restricted to units without routine hourly monitoring (complete-case N = 953), no hospital- or ICU-level characteristic remained significant after FDR adjustment (Fig. 3C). Results of the full-sample escalation sensitivity analysis are shown in Supplementary Table S5.

**Table 4:** Multivariable determinants of routine hourly urine output monitoring, hourly monitoring during AKI, and escalation of monitoring frequency during AKI.

Characteristic	Level	(A) Routine hourly UO monitoring (N = 1,888)		(B) Hourly UO monitoring during AKI (N = 1,888)		(C) Escalation during AKI among ICUs without routine hourly monitoring (N = 953)	
		aOR (95% CI)	q value	aOR (95% CI)	q value	aOR (95% CI)	q value
Hospital level	Tertiary	Ref		Ref		Ref	
	Non-tertiary	1.09 (0.84–1.40)	0.737	0.83 (0.60–1.15)	0.520	0.73 (0.49–1.09)	0.378
Teaching hospital	No	Ref		Ref		Ref	
	Yes	1.04 (0.84–1.28)	0.816	1.20 (0.91–1.59)	0.485	1.35 (0.96–1.91)	0.378
Hospital type	General	Ref		Ref		Ref	
	Specialty	0.80 (0.53–1.20)	0.407	1.09 (0.65–1.81)	0.952	1.49 (0.76–2.93)	0.509
ICU type	General ICU	Ref		Ref		Ref	
	Specialised ICU	0.77 (0.56–1.07)	0.239	0.86 (0.56–1.30)	0.715	0.85 (0.51–1.43)	0.738
Region	East China	Ref		Ref		Ref	
	North China	1.38 (1.02–1.87)	0.105	0.94 (0.62–1.44)	0.952	0.66 (0.39–1.10)	0.378
	Northeast China	1.54 (1.10–2.15)	0.071	0.75 (0.48–1.19)	0.485	0.48 (0.28–0.82)	0.132
	Central China	0.94 (0.61–1.45)	0.816	0.60 (0.34–1.05)	0.485	0.90 (0.43–1.88)	0.865
	South China	**2.13 (1.59–2.87)**	**<0.001**	1.00 (0.65–1.55)	0.985	0.96 (0.54–1.71)	0.902
	Southwest China	1.08 (0.80–1.47)	0.779	1.39 (0.92–2.11)	0.485	0.87 (0.52–1.45)	0.738
	Northwest China	0.79 (0.55–1.12)	0.301	0.76 (0.50–1.17)	0.485	0.58 (0.35–0.95)	0.305
ICU beds	≤10	Ref		Ref		Ref	
	11–15	1.02 (0.77–1.37)	0.868	0.99 (0.69–1.43)	0.985	1.19 (0.76–1.89)	0.652
	16–23	1.39 (1.01–1.92)	0.105	0.82 (0.54–1.24)	0.577	0.70 (0.42–1.15)	0.378
	≥24	1.45 (1.00–2.10)	0.115	1.05 (0.63–1.75)	0.952	0.62 (0.33–1.18)	0.378
Annual ICU admissions	≤330	Ref		Ref		Ref	
	331–568	1.24 (0.94–1.64)	0.240	1.06 (0.74–1.51)	0.952	1.21 (0.79–1.88)	0.637
	569–956	1.44 (1.05–1.97)	0.098	1.37 (0.91–2.05)	0.485	1.11 (0.68–1.82)	0.789
	≥957	1.48 (1.03–2.13)	0.105	1.40 (0.85–2.30)	0.485	1.72 (0.92–3.22)	0.378
Nurse-to-bed ratio	<2.5	Ref		Ref		Ref	
	2.5 to <3.0	**1.61 (1.17–2.22)**	**0.034**	1.26 (0.78–2.04)	0.577	0.75 (0.43–1.33)	0.628
	≥3.0	1.09 (0.69–1.73)	0.816	0.94 (0.50–1.74)	0.952	0.73 (0.35–1.53)	0.637
Routine UO monitoring frequency	Every ≥6 h/irregular	—	—	Ref		Ref	
	Every 2–4 h	—	—	**1.85 (1.42–2.41)**	**<0.001**	0.98 (0.71–1.33)	0.902
	Every 1 h	—	—	**42.06 (27.10–65.30)**	**<0.001**	NA	NA

Data are aORs with 95% CIs. All models were mutually adjusted for hospital level, teaching status, hospital type, ICU type, region, ICU beds, annual ICU admissions, and nurse-to-bed ratio.

Model A evaluated routine 1 -h UO monitoring; Model B evaluated 1 -h UO monitoring during AKI and additionally adjusted for routine UO monitoring frequency; Model C evaluated escalation during AKI among ICUs without routine hourly monitoring.

For Model C, escalation was a shorter scheduled interval during AKI; conversion from irregular routine monitoring to any scheduled interval was counted as escalation. Complete-case analysis was used. q values were calculated separately within each model using the Benjamini–Hochberg FDR method; bold indicates q<0.05.

Physician and nurse headcounts were excluded because of collinearity; nursing intensity was represented by the nurse-to-bed ratio. Abbreviations: AKI, acute kidney injury; aOR, adjusted odds ratio; CI, confidence interval; FDR, false discovery rate; ICU, intensive care unit; NA, not applicable; UO, urine output.

**Fig. 3:**
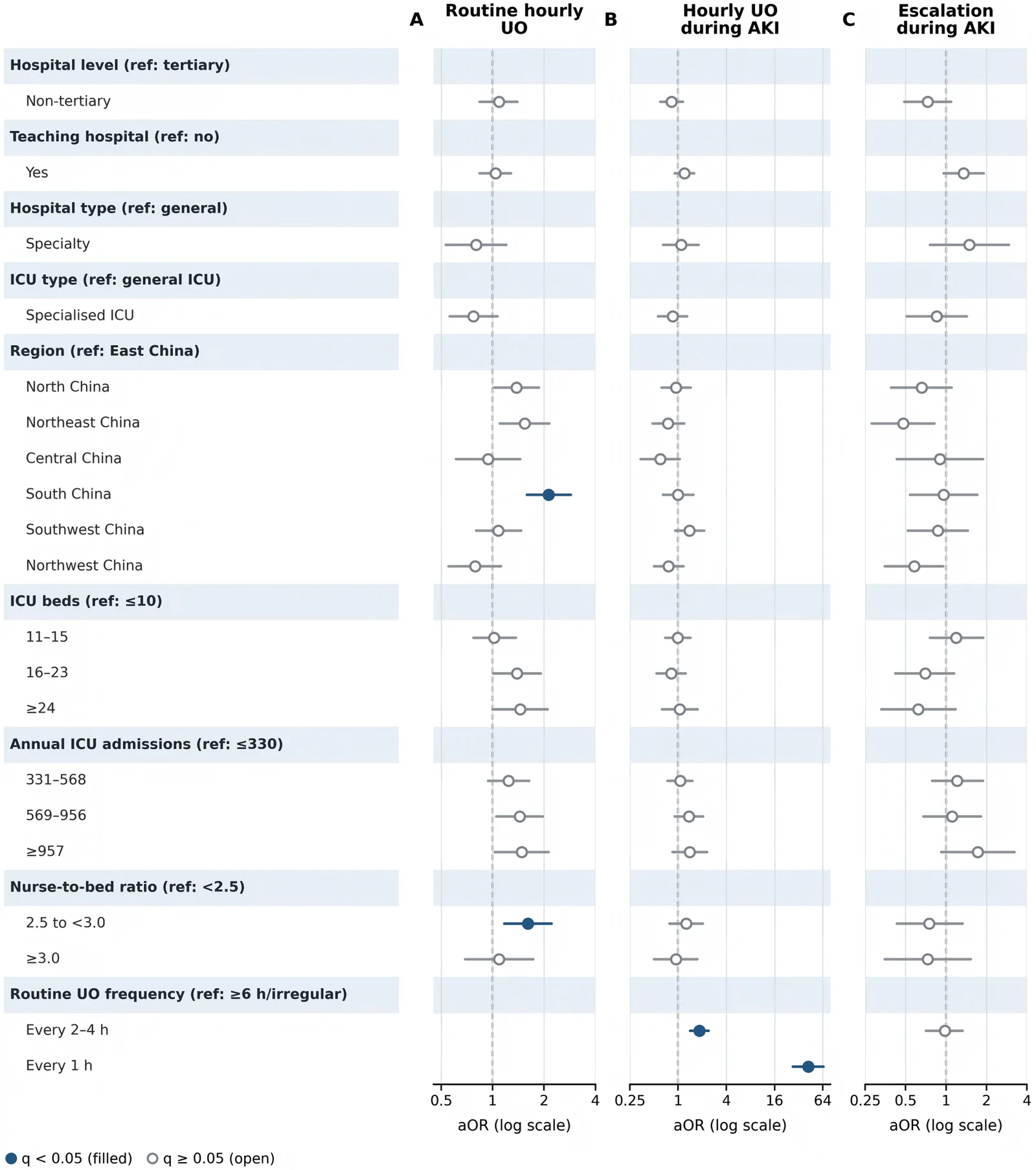
Multivariable associations with urine-output monitoring practices. Forest plots show adjusted odds ratios (aORs) and 95% confidence intervals for (A) routine hourly urine output (UO) monitoring (N = 1,888), (B) hourly UO monitoring during AKI (N = 1,888), and (C) escalation of UO monitoring during AKI among ICUs without routine hourly monitoring (N = 953). Reference categories are specified in the variable headings. The dashed vertical line indicates an aOR of 1.0. Filled symbols indicate q < 0.05 after Benjamini–Hochberg false discovery rate correction within each model; open symbols indicate q ≥ 0.05. Models B and C included routine UO-monitoring frequency as a covariate. The “Every 1 h” category was not applicable to Model C because this analysis was restricted to ICUs without routine hourly monitoring. AKI, acute kidney injury; aOR, adjusted odds ratio; ICU, intensive care unit; UO, urine output.

As an exploratory secondary analysis, AKI-MIS was calculated for all 1,896 ICU units using the scoring framework in Supplementary Table S7. The median score was 68.1 (IQR, 60.3–74.9), with a range of 19.1–100.0 (Fig. 4A). In the multivariable analysis of 1,888 complete cases, teaching hospitals had a 2.36-point higher adjusted mean AKI-MIS than non-teaching hospitals (95% CI, 1.16–3.56; FDR-adjusted q = 0.002). No other hospital- or ICU-level characteristic remained significant after FDR adjustment (Fig. 4B).

**Fig. 4:**
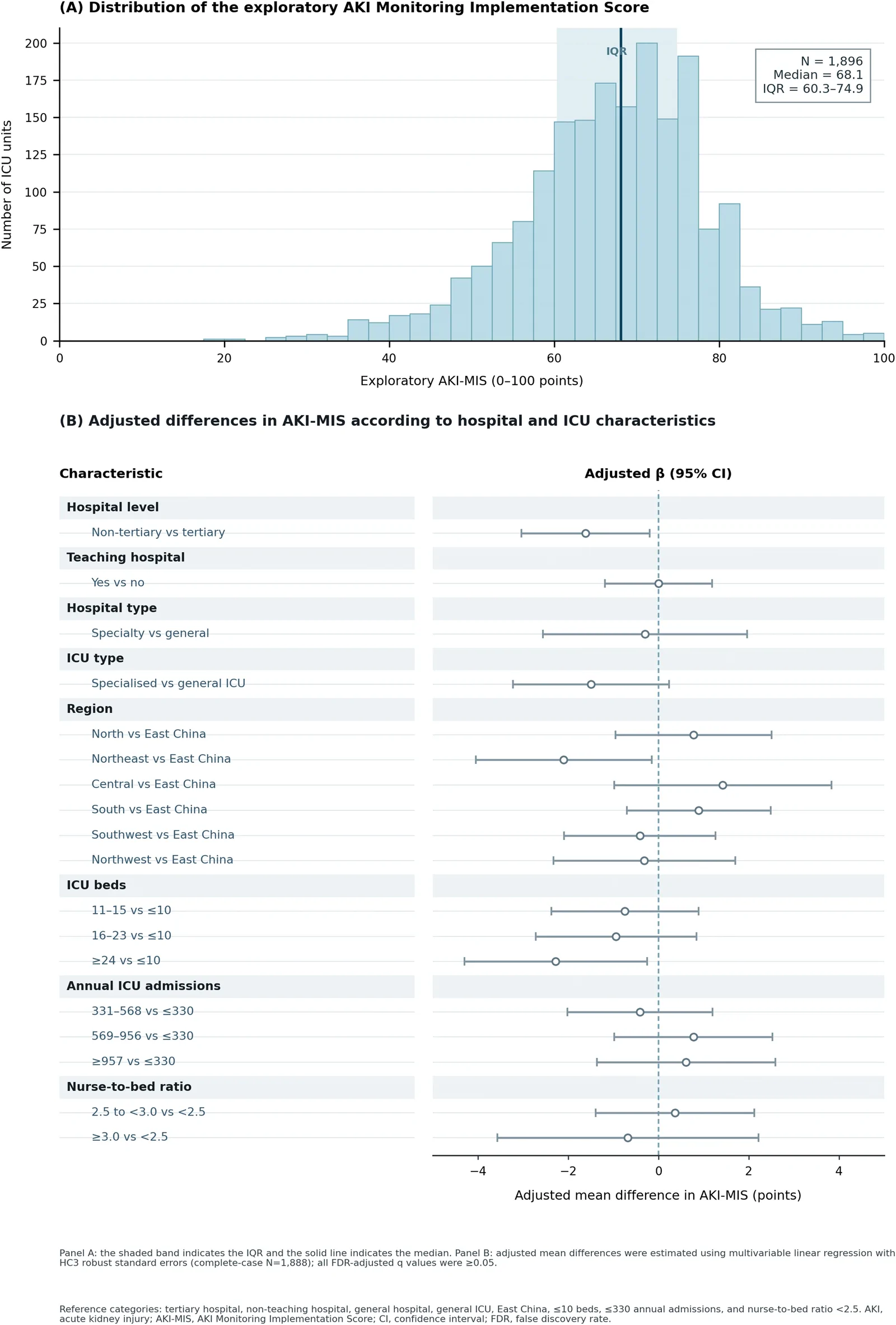
Distribution and multivariable determinants of the exploratory AKI Monitoring Implementation Score. (A) Distribution of AKI-MIS among 1,896 participating adult ICU units. The shaded band indicates the IQR, and the solid vertical line indicates the median. (B) Adjusted mean differences in AKI-MIS according to hospital and ICU characteristics, estimated using multivariable ordinary least-squares regression with HC3 robust standard errors (complete-case N = 1,888). Horizontal lines indicate 95% CIs; the dashed vertical line indicates no adjusted difference. FDR-adjusted q values were calculated using the Benjamini–Hochberg method; filled symbols indicate q < 0.05. Reference categories were tertiary hospital, non-teaching hospital, general hospital, general ICU, East China, ≤10 ICU beds, ≤330 annual ICU admissions, and nurse-to-bed ratio <2.5. AKI, acute kidney injury; AKI-MIS, AKI Monitoring Implementation Score; CI, confidence interval; FDR, false discovery rate; ICU, intensive care unit.

The alternative non-domain-equalized weighting correlated closely with the primary AKI-MIS (Spearman’s ρ = 0.981). Teaching-hospital status remained associated with a higher score (adjusted mean difference, 2.33 points; 95% CI, 1.05–3.61; FDR-adjusted q = 0.007), and the overall coefficient pattern was similar (Supplementary Table S8).

## Discussion

In this large nationwide survey of 1,896 adult ICU units across all 31 provincial-level administrative regions of mainland China, AKI diagnosis and monitoring were widely reported, but implementation remained uneven across staging, bedside UO surveillance, operational definitions, and digital integration. Nearly all units reported diagnosing AKI (97.9%) and 75.9% considered both UO and SCr, whereas only 63.2% explicitly implemented AKI staging. Correlates differed by outcome: baseline UO-monitoring frequency was strongly associated with hourly monitoring during AKI, whereas South China and a nurse-to-bed ratio of 2.5 to <3.0 were associated with routine hourly monitoring. No measured characteristic was associated with escalation among units without routine hourly monitoring after FDR correction, and hospital scale was not independently associated with these outcomes. The exploratory AKI-MIS had a median of 68.1, and teaching-hospital status was the only measured characteristic associated with a higher score after FDR correction. These findings shift the practical focus from whether AKI is recognised to how consistently monitoring is standardised, intensified, and linked to clinical response.

### Detection and surveillance structure

AKI recognition was widespread at the ICU-unit level, but the surveillance structure underpinning recognition was not equally mature across its components. Although 97.9% of ICU units reported diagnosing AKI and 75.9% reported approximately equal reliance on UO and SCr, only 63.2% explicitly implemented AKI staging. SCr monitoring appeared structurally more stable, with 70.7% of units already achieving at-least-daily monitoring under routine practice and 92.3% and 91.8%, respectively, doing so in AKI and sepsis-associated AKI. By contrast, UO monitoring remained more dependent on bedside workflow, with routine hourly monitoring reported by 49.6% of units and irregular monitoring still reported by 8.0%.

This distinction is clinically important because UO is not simply a supporting variable; it is a core physiologic signal for AKI recognition and risk stratification. A systematic review by Malbrain et al. showed that incorporation of UO criteria provides substantial diagnostic and prognostic information and often identifies AKI earlier than SCr alone [29]. Ralib et al. further showed that UO-based AKI classification is highly sensitive to the thresholds and time windows applied [30]. These findings suggest that many participating ICUs have adopted a dual-marker framework for AKI recognition, but that UO surveillance remains more workflow-dependent than laboratory-driven SCr monitoring and is therefore more variable in practice.

### Monitoring intensity, transitions, and implementation capacity

Reported monitoring frequency increased in higher-risk clinical scenarios. Hourly UO monitoring increased from 49.6% under routine practice to 74.1% in sepsis, 75.0% in AKI, and 77.7% in sepsis-associated AKI; at-least-daily SCr monitoring increased from 70.7% under routine practice to 92.3% in AKI and 91.8% in sepsis-associated AKI. Among units already monitoring UO hourly in routine practice, 97.3% remained hourly during AKI. Among units without routine hourly monitoring, 76.7% moved to a shorter scheduled interval or from irregular to scheduled monitoring during AKI. This paired transition analysis distinguishes maintenance of an already intensive practice from true escalation. Consistently, baseline UO-monitoring intensity was the dominant correlate of hourly monitoring during AKI in Model B.

The findings also reveal a gap between clinical support for frequent monitoring and the capacity to deliver it. Although 93.2% of respondents believed that increased UO monitoring facilitates earlier AKI recognition and 91.2% considered hourly monitoring feasible in patients with sepsis, 83.5% anticipated increased nursing workload and 69.9% identified workload or insufficient staffing as the main barrier. In Model A, South China and a nurse-to-bed ratio of 2.5 to <3.0 were associated with routine hourly monitoring after FDR correction. However, among units without routine hourly monitoring, no measured hospital- or ICU-level characteristic remained associated with escalation after FDR correction. Region should therefore be interpreted as a composite contextual marker rather than a causal exposure, and the subgroup result suggests that unmeasured local workflows may be more important for escalation than the structural characteristics captured by the survey.

Sepsis-associated AKI evolves dynamically, making timely reassessment particularly important [31]. Sustained high-intensity surveillance, however, is influenced by staffing and workload, and ICU workforce studies have linked patient outcomes with staff resources and nurse workload [32,33]. Our results extend this concern to a care process, while also refining its interpretation: the nurse-to-bed association applied to routine hourly monitoring, not to escalation within the non-hourly subgroup. Neither ICU-bed category nor annual ICU-admission category remained significant after FDR correction, so the data do not support an independent J-shaped scale or volume effect. Moreover, annual ICU admissions are not equivalent to annual AKI case volume, which was not collected.

### Standardisation and guideline concordance

A further key finding was the marked heterogeneity in how UO monitoring was operationalised. Among units using dynamic UO monitoring, 76.8% recorded output in mL/h and 23.2% in mL/kg/h; among units using manual monitoring, 78.2% recorded UO in mL/h, 4.1% in mL/kg/h, and 17.7% as absolute volume in mL. Threshold definitions were likewise heterogeneous. Of the 131 units using weight-based thresholds, 42 (32.1%) used the KDIGO stage 1 definition of <0.5 mL/kg/h for 6 hours. Of the 1,479 units using mL/h thresholds, 92.2% used <30 mL/h, although duration criteria varied; a further 286 units used absolute-volume thresholds. Thus, the same clinical construct—oliguria—was operationalised through multiple bedside rule sets.

This heterogeneity matters in practice as well as in documentation. It can influence when AKI is recognised, how consistently staging criteria are applied, and how comparable monitoring data are across centres. Ralib et al. demonstrated that UO-based AKI classification is highly sensitive to the exact threshold and time-window definitions used. More recent multicentre work has likewise shown that large-scale use of UO data is strongly affected by variation in charting and rule implementation when standardisation is lacking. Our findings therefore suggest that different ICUs have translated the same guideline concepts into different bedside rules. Local flexibility may facilitate implementation, but clearer minimum definitions are needed for consistent recognition, escalation, and cross-unit comparison.

### Biomarkers, digitalisation, and the response loop

Digital and advanced AKI monitoring had been adopted in some ICUs, but integration remained incomplete. Only 14.8% of units reported dynamic UO monitoring and 26.0% reported an AKI high-risk early-warning system. Among dynamically monitored units, 96.1% reported physician alerts for oliguria, whereas 55.0% reported upload of the corresponding data to the EMR. Biomarker use was dominated by cystatin C (70.4%); NGAL was reported by 17.4%, and all other named biomarkers by fewer than 15% of units. Within the six prespecified named biomarker fields, cystatin C as the only selected named biomarker was the most common pattern (50.3%), followed by NGAL plus cystatin C (6.8%) and interleukin-18 plus cystatin C (5.9%). Regional variation remained significant after FDR correction for NGAL and “Other” biomarkers. These findings describe reported availability and combinations, but the survey did not collect the clinical indications for biomarker testing and therefore cannot distinguish screening, diagnostic, prognostic, or research use.

The practical value of digital tools depends not only on their availability, but also on whether they are embedded in a usable clinical workflow. ADQI consensus statements on electronic AKI detection emphasise that automated alerts should be linked to actionable recommendations and care pathways rather than function merely as diagnostic labels [34]. A recent systematic review and meta-analysis similarly suggested that electronic AKI alerts may improve care processes and reduce AKI progression, although mortality benefits remain inconsistent [35]. The ADQI digital health consensus further emphasises linking monitoring, interpretation, and action [36]. Our findings fit this view: individual digital components were more common than a dependable closed loop from signal detection to bedside response. The observed regional biomarker differences should remain descriptive because region may capture multiple differences in resources, laboratory access, case mix, and local practice that were not measured directly.

### Exploratory AKI monitoring implementation score

The investigator-developed AKI-MIS is an exploratory formative composite describing the breadth of AKI-monitoring processes rather than a validated measure of care quality. The median score was 68.1, and teaching hospitals had a 2.36-point higher adjusted mean score than non-teaching hospitals; no other characteristic remained significant after FDR correction. Alternative weighting produced highly similar scores (Spearman’s ρ = 0.981) and retained the teaching-hospital association. However, this numerical robustness does not establish construct or predictive validity. Given the low explained variance and absence of patient-level outcome validation, AKI-MIS should be used descriptively rather than to rank ICUs, pending external validation.

### Strengths, limitations, and implications

Strengths of this study include its broad geographic coverage, large ICU-unit sample, multidimensional assessment of AKI monitoring, and paired analysis of routine-to-AKI transitions. Prespecified data-quality checks, FDR correction, collinearity assessment, sensitivity analysis, and alternative AKI-MIS weighting further strengthened the analysis.

Several limitations should be acknowledged. Professional-network dissemination used non-probability sampling; therefore, the invitation denominator, response rate, and comparisons with non-participating ICUs were unavailable, and tertiary and teaching hospitals predominated. The estimates consequently describe participating units rather than nationally representative prevalence. Data were self-reported, respondent identity was not independently verified, and anonymous collection precluded hospital-level clustering. Because each ICU unit was represented by a single respondent, responses may have been affected by social-desirability bias, recall error, and subjective interpretation of terms such as “hourly monitoring,” and may not capture within-unit variation in practice. The cross-sectional design provides only a snapshot of reported practice, which may change over time; moreover, the absence of patient-level data prevents confirmation that reported ICU practices reflected actual care and precludes causal or outcome inference. Annual AKI case volume and biomarker-testing indications were not collected. In addition, units already monitoring hourly could not further escalate monitoring frequency, and AKI-MIS remains exploratory and unvalidated.

Recent national surveys have documented substantial geographic and institutional variation in Chinese critical care resources [37,38]. Priorities therefore include standardised oliguria definitions and AKI staging, workflow-compatible monitoring escalation, and digital pathways linking abnormal findings to clinical action. Regional associations should be interpreted contextually rather than causally, and prospective studies should determine whether these improvements enhance patient outcomes.

## Conclusion

Among participating adult ICU units across all 31 provincial-level administrative regions of mainland China, AKI diagnosis and monitoring were widely reported, but substantial heterogeneity remained across monitoring systems. These findings provide a broad descriptive benchmark and identify priorities for strengthening standardisation, risk-responsive escalation, and closed-loop response pathways.

## Contributors

Xinran Wang, Mingyan Zhao, Kaijiang Yu, and Changsong Wang conceived and designed the study. Xinran Wang, Xiuhua Zhang, Weiting Zhang, Yu Xin, Pengfei Huang, Yanqi Liu, Kai Zeng, and Xianglin Meng contributed to questionnaire development, data collection, and data curation. Zhiyong Peng, Wenxiong Li, Zhenjie Hu, Yingzi Huang, and Meili Duan contributed to survey coordination and critical review of the study design. Xinran Wang directly accessed and verified the underlying data and did the statistical analysis. Mingyan Zhao, Kaijiang Yu, and Changsong Wang also directly accessed and verified the underlying data. Xinran Wang drafted the manuscript. All authors contributed to data interpretation, critically revised the manuscript for important intellectual content, had full access to all the data in the study, and had final responsibility for the decision to submit for publication.

## Consent for publication

Not applicable.

## Ethical approval and consent to participate

The Ethics Committee of the First Affiliated Hospital of Harbin Medical University determined that this anonymous questionnaire survey of ICU units, which collected no patient information and no hospital identifiers, was exempt from ethics review. Participation was voluntary, and completion of the questionnaire was taken as consent to participate.

## Role of the funding source

This study was supported by the 10.13039/501100001809National Natural Science Foundation of China (Nos. 82472198, 82672824, and 82430069).

## Data sharing

The questionnaire, prespecified scoring framework for AKI-MIS, and aggregated data supporting the findings of this study are available from the corresponding authors upon reasonable request. Because the survey was anonymous and conducted at the ICU-unit level, no patient-level data were collected.

## Declaration of competing interest

We declare no competing interests.

The authors declare that they have no competing interests.
