# Supplementary material for: National variation in acute kidney injury diagnosis and monitoring across adult intensive care units in china: a nationwide cross-sectional survey
Source: Ann Intensive Care. 2026 Sep 22;16:100166. doi: 10.1016/j.aicoj.2026.100166 (PMC13673355; doi:10.1016/j.aicoj.2026.100166)
Supplement: Supplementary file 1 [file mmc1.docx]

**Supplementary Table S1. Province-level distribution of participating adult ICU units and contextual comparison with a published national critical care resource survey**

| **Region** | **Province** | **Current survey: participating adult ICU units, 2025 n (% of N=1,896)** | **Published national resource survey: hospitals with a comprehensive ICU, 2021-2023 n (% of N=4,744)** |
| --- | --- | --- | --- |
| North China | Beijing | 89 (4.7%) | 70 (1.5%) |
| North China | Hebei | 63 (3.3%) | 214 (4.5%) |
| North China | Inner Mongolia | 39 (2.1%) | 94 (2.0%) |
| North China | Shanxi | 32 (1.7%) | 106 (2.2%) |
| North China | Tianjin | 19 (1.0%) | 54 (1.1%) |
| Northeast China | Heilongjiang | 65 (3.4%) | 87 (1.8%) |
| Northeast China | Jilin | 36 (1.9%) | 39 (0.8%) |
| Northeast China | Liaoning | 97 (5.1%) | 117 (2.5%) |
| East China | Anhui | 152 (8.0%) | 184 (3.9%) |
| East China | Fujian | 36 (1.9%) | 146 (3.1%) |
| East China | Jiangsu | 133 (7.0%) | 231 (4.9%) |
| East China | Jiangxi | 155 (8.2%) | 181 (3.8%) |
| East China | Shandong | 162 (8.5%) | 300 (6.3%) |
| East China | Shanghai | 4 (0.2%) | 88 (1.9%) |
| East China | Zhejiang | 13 (0.7%) | 283 (6.0%) |
| Central China | Henan | 78 (4.1%) | 332 (7.0%) |
| Central China | Hubei | 15 (0.8%) | 150 (3.2%) |
| Central China | Hunan | 10 (0.5%) | 188 (4.0%) |
| South China | Guangdong | 155 (8.2%) | 395 (8.3%) |
| South China | Guangxi | 120 (6.3%) | 193 (4.1%) |
| South China | Hainan | 8 (0.4%) | 33 (0.7%) |
| Southwest China | Chongqing | 41 (2.2%) | 106 (2.2%) |
| Southwest China | Guizhou | 19 (1.0%) | 178 (3.8%) |
| Southwest China | Sichuan | 99 (5.2%) | 357 (7.5%) |
| Southwest China | Tibet | 1 (0.1%) | 10 (0.2%) |
| Southwest China | Yunnan | 81 (4.3%) | 179 (3.8%) |
| Northwest China | Gansu | 15 (0.8%) | 103 (2.2%) |
| Northwest China | Ningxia | 26 (1.4%) | 33 (0.7%) |
| Northwest China | Qinghai | 28 (1.5%) | 22 (0.5%) |
| Northwest China | Shaanxi | 27 (1.4%) | 141 (3.0%) |
| Northwest China | Xinjiang | 78 (4.1%) | 130 (2.7%) |
| **Total** |  | **1,896 (100.0%)** | **4,744 (100.0%)** |

**Note.** Data are n (% of the respective survey total). The current study used the adult ICU unit as the survey and analysis unit. The external benchmark used the hospital with a comprehensive ICU as its unit and was derived from Table 2 of Zhang X, et al., Critical care resource disparities in China: a nationwide survey and policy recommendations for health equity. Critical Care. 2026;30:209. doi:10.1186/s13054-026-05983-1. Because the two surveys used different units, periods, eligibility criteria, and recruitment procedures, the comparison is descriptive only. The external hospital counts were not used as denominators for response rates, hospital coverage, weighting, or ICU-per-hospital ratios. Hospital identifiers and a national sampling frame of eligible adult ICU units were unavailable in the current survey. ICU, intensive care unit.

**Supplementary Table S2. Oliguria alert threshold formats and criteria used in participating adult ICU units**

| **Panel A. Threshold reporting format in the full analytic sample (N = 1,896)** | | | |
| --- | --- | --- | --- |
| **Threshold format** | **Description** | **n** | **%** |
| mL/kg/h | Weight-normalized rate threshold | 131 | 6.9% |
| mL/h | Absolute hourly rate threshold | 1,479 | 78.0% |
| mL | Absolute urine-volume threshold (non-rate) | 286 | 15.1% |
| Missing threshold-format classification | - | 0 | 0.0% |
| **Panel B. Detailed criteria among units reporting mL/kg/h thresholds (N = 131)** | | | |
| **Cutoff** | **Duration** | **n** | **%** |
| <0.5 mL/kg/h | 12 h | 32 | 24.4% |
| <0.5 mL/kg/h | 6 h | 42 | 32.1% |
| <0.5 mL/kg/h | 3 h | 22 | 16.8% |
| <0.5 mL/kg/h | 2 h | 18 | 13.7% |
| <0.5 mL/kg/h | 1 h | 7 | 5.3% |
| <0.3 mL/kg/h | 12 h | 1 | 0.8% |
| <0.3 mL/kg/h | 6 h | 4 | 3.1% |
| <0.3 mL/kg/h | 3 h | 2 | 1.5% |
| <0.3 mL/kg/h | 2 h | 2 | 1.5% |
| <0.3 mL/kg/h | 1 h | 1 | 0.8% |
| **Panel C. Detailed criteria among units reporting mL/h thresholds (N = 1,479)** | | | |
| **Cutoff** | **Duration** | **n** | **%** |
| <30 mL/h | 12 h | 75 | 5.1% |
| <30 mL/h | 6 h | 329 | 22.2% |
| <30 mL/h | 3 h | 348 | 23.5% |
| <30 mL/h | 2 h | 346 | 23.4% |
| <30 mL/h | 1 h | 265 | 17.9% |
| <18 mL/h | 12 h | 4 | 0.3% |
| <18 mL/h | 6 h | 16 | 1.1% |
| <18 mL/h | 3 h | 15 | 1.0% |
| <18 mL/h | 2 h | 22 | 1.5% |
| <18 mL/h | 1 h | 36 | 2.4% |
| Other explicit threshold | - | 23 | 1.6% |

Panel A includes all 1,896 ICU units; threshold-format categories are mutually exclusive and sum to the full analytic sample. Percentages in Panel A use N = 1,896, whereas percentages in Panels B and C use the corresponding panel denominator. To avoid double counting, the dynamic-monitoring branch was used when available; otherwise, the manual-monitoring branch was used. The 286 units selecting mL reported non-rate absolute-volume thresholds collected as free text; these units are shown in Panel A but are not included in the rate-based detailed Panels B and C. Missing threshold-format classification: n = 0. Threshold options in Panels B and C reflect the questionnaire response categories. ICU, intensive care unit.

**Supplementary Table S3. Perceptions, feasibility, and barriers related to intensified urine output monitoring**

| **Panel A. Perceived benefits, feasibility, workload, and support** | | |
| --- | --- | --- |
| **Item** | **Available N** | **Result** |
| Increasing urine output monitoring frequency helps early AKI diagnosis | 1,896 | 1,768 (93.2%) |
| Hourly urine output monitoring is feasible in patients with sepsis | 1,896 | 1,730 (91.2%) |
| Hourly urine output monitoring is expected to increase nursing workload | 1,896 | 1,583 (83.5%) |
| Manual urine output measurement is affected by subjective and/or objective factors | 1,783 | 1,244 (69.8%) |
| Support score for hourly monitoring in sepsis-associated AKI (1-10 points) | 1,896 | 10 (8-10) |
| **Panel B. Main implementation challenge (single-choice item)** | | |
| **Item** | **Available N** | **Result** |
| Increased nursing workload / insufficient staffing | 1,896 | 1,325 (69.9%) |
| Additional cost (equipment / medical expenditure) | 1,896 | 452 (23.8%) |
| Other | 1,896 | 119 (6.3%) |

Data are n (%) unless otherwise indicated. The available denominator is shown for each item. The manual-measurement item applied to 1,783 ICU units; 113 units were coded as not applicable according to questionnaire routing and were excluded from that denominator. All other items had complete responses (N = 1,896). The main-challenge item was single choice, and its categories sum to 1,896. Although the questionnaire displayed an “Other” write-in field, the exported analytic dataset did not retain a separately analysable text variable; therefore, these responses could not be further classified. The support score is presented as median (IQR). AKI, acute kidney injury; ICU, intensive care unit.

**Supplementary Table S4. Missing-data and analytic-sample accounting for the main and supplementary analyses**

**Panel A. Analysis-level sample accounting**

| **Analysis** | **Eligible/source N** | **Complete/available N** | **Excluded for missing covariates, n** | **Structurally ineligible, n** |
| --- | --- | --- | --- | --- |
| Descriptive analyses | 1,896 | 1,896 | 0 | 0 |
| Model A: routine hourly UO monitoring | 1,896 | 1,888 | 8 | 0 |
| Model B: hourly UO monitoring during AKI | 1,896 | 1,888 | 8 | 0 |
| Model C: escalation during AKI among ICUs without routine hourly monitoring | 956 | 953 | 3 | 940 |
| Model D: full-sample escalation sensitivity analysis | 1,896 | 1,888 | 8 | 0 |
| AKI-MIS score construction and weighting comparison | 1,896 | 1,896 | 0 | 0 |
| AKI-MIS regression (primary and alternative scores) | 1,896 | 1,888 | 8 | 0 |

Models A, B, and D and both AKI-MIS regression specifications excluded the same 8 records with incomplete core covariates. Model C was restricted to 956 ICUs without routine hourly monitoring; 940 routine-hourly ICUs were structurally ineligible, and 3 of the 956 eligible ICUs had incomplete core covariates.

**Panel B. Item-level missingness and structural non-applicability**

| **Variable (analysis label)** | **Applicable N** | **Observed, n** | **Missing, n** | **Missing, %** | **Structurally N/A, n** |
| --- | --- | --- | --- | --- | --- |
| **Main and supplementary outcomes** | | | | | |
| Routine UO monitoring frequency | 1,896 | 1,896 | 0 | 0.0% | 0 |
| Routine hourly UO monitoring (derived Model A outcome) | 1,896 | 1,896 | 0 | 0.0% | 0 |
| UO monitoring frequency during AKI | 1,896 | 1,896 | 0 | 0.0% | 0 |
| Hourly UO monitoring during AKI (derived Model B outcome) | 1,896 | 1,896 | 0 | 0.0% | 0 |
| Escalation during AKI (derived Models C/D outcome) | 1,896 | 1,896 | 0 | 0.0% | 0 |
| **Geography and institutional characteristics** | | | | | |
| Province | 1,896 | 1,896 | 0 | 0.0% | 0 |
| Region (7 major regions) | 1,896 | 1,896 | 0 | 0.0% | 0 |
| Hospital level (tertiary vs non-tertiary) | 1,896 | 1,896 | 0 | 0.0% | 0 |
| Teaching hospital | 1,896 | 1,896 | 0 | 0.0% | 0 |
| Hospital type | 1,896 | 1,896 | 0 | 0.0% | 0 |
| ICU type | 1,896 | 1,896 | 0 | 0.0% | 0 |
| **Resource variables and derived categories** | | | | | |
| ICU beds and 4-level category | 1,896 | 1,895 | 1 | 0.1% | 0 |
| Annual ICU admissions and 4-level category | 1,896 | 1,889 | 7 | 0.4% | 0 |
| Nurse-to-bed ratio and 3-level category | 1,896 | 1,894 | 2 | 0.1% | 0 |
| ICU physicians (supplementary correlation analysis) | 1,896 | 1,893 | 3 | 0.2% | 0 |
| ICU nurses (supplementary correlation analysis and ratio derivation) | 1,896 | 1,894 | 2 | 0.1% | 0 |
| **AKI detection and serum creatinine monitoring** | | | | | |
| AKI diagnosis performed | 1,896 | 1,896 | 0 | 0.0% | 0 |
| AKI staging implemented | 1,896 | 1,896 | 0 | 0.0% | 0 |
| Primary basis for AKI diagnosis | 1,896 | 1,896 | 0 | 0.0% | 0 |
| AKI high-risk early-warning system | 1,896 | 1,896 | 0 | 0.0% | 0 |
| eGFR routinely calculated | 1,896 | 1,896 | 0 | 0.0% | 0 |
| Routine sCr monitoring frequency | 1,896 | 1,896 | 0 | 0.0% | 0 |
| sCr monitoring frequency in sepsis | 1,896 | 1,896 | 0 | 0.0% | 0 |
| sCr monitoring frequency in AKI | 1,896 | 1,896 | 0 | 0.0% | 0 |
| sCr monitoring frequency in SA-AKI | 1,896 | 1,896 | 0 | 0.0% | 0 |
| **Urine output monitoring and oliguria criteria** | | | | | |
| UO monitoring frequency in sepsis | 1,896 | 1,896 | 0 | 0.0% | 0 |
| UO monitoring frequency in SA-AKI | 1,896 | 1,896 | 0 | 0.0% | 0 |
| UO monitoring adjusted by clinical severity | 1,896 | 1,896 | 0 | 0.0% | 0 |
| sCr monitoring adjusted by clinical severity | 1,896 | 1,896 | 0 | 0.0% | 0 |
| UO monitoring mode | 1,896 | 1,896 | 0 | 0.0% | 0 |
| Oliguria threshold format used in revised Table S2 | 1,896 | 1,896 | 0 | 0.0% | 0 |
| **Perceptions, feasibility, and barriers** | | | | | |
| Higher-frequency UO monitoring helps early AKI diagnosis | 1,896 | 1,896 | 0 | 0.0% | 0 |
| Hourly UO monitoring increases nursing workload | 1,896 | 1,896 | 0 | 0.0% | 0 |
| Hourly UO monitoring is feasible in patients with sepsis | 1,896 | 1,896 | 0 | 0.0% | 0 |
| Main implementation challenge | 1,896 | 1,896 | 0 | 0.0% | 0 |
| Support score for hourly monitoring in SA-AKI | 1,896 | 1,896 | 0 | 0.0% | 0 |
| Manual UO measurement affected by subjective/objective factors | 1,783 | 1,783 | 0 | 0.0% | 113 |
| **Exploratory AKI Monitoring Implementation Score** | | | | | |
| Primary equal-domain AKI-MIS | 1,896 | 1,896 | 0 | 0.0% | 0 |
| Alternative raw-point-weighted AKI-MIS | 1,896 | 1,896 | 0 | 0.0% | 0 |

Missingness denotes empty/NA values; percentages use the applicable denominator shown. Structural non-applicability arose from questionnaire routing or analytic eligibility and was not treated as missing. The manual-measurement perception item applied to 1,783 ICUs; 113 were coded not applicable. Derived categories inherited missingness from their source variables. The 8 complete-case exclusions reflect the union of missing core covariates (ICU beds, annual ICU admissions, and nurse-to-bed ratio) and therefore are not the sum of item-specific missing counts. Physician and nurse headcounts were used only in the supplementary resource-correlation analysis and were excluded from the final regression models because of collinearity. AKI-MIS component responses were handled according to the prespecified branch and not-classifiable scoring rules in Supplementary Table S7; no statistical imputation was performed. AKI, acute kidney injury; AKI-MIS, AKI Monitoring Implementation Score; eGFR, estimated glomerular filtration rate; ICU, intensive care unit; N/A, not applicable; SA-AKI, sepsis-associated AKI; sCr, serum creatinine; UO, urine output.

**Supplementary Table S5. Sensitivity analysis of multivariable determinants of escalation of urine output monitoring frequency during AKI in the full sample**

| **Characteristic** | **Level** | **aOR (95% CI)** | **P value** | **FDR-adjusted q value** |
| --- | --- | --- | --- | --- |
| **Hospital level** | Tertiary | Ref |  |  |
|  | Non-tertiary | 0.83 (0.64–1.08) | 0.158 | 0.316 |
| **Teaching hospital** | No | Ref |  |  |
|  | Yes | 1.11 (0.89–1.38) | 0.360 | 0.589 |
| **Hospital type** | General | Ref |  |  |
|  | Specialty | 1.39 (0.93–2.06) | 0.108 | 0.278 |
| **ICU type** | General ICU | Ref |  |  |
|  | Specialised ICU | 1.17 (0.85–1.62) | 0.342 | 0.589 |
| **Region** | East China | Ref |  |  |
|  | North China | **0.66 (0.48–0.91)** | **0.011** | **0.047** |
|  | Northeast China | **0.53 (0.37–0.75)** | **<0.001** | **0.004** |
|  | Central China | 1.01 (0.65–1.55) | 0.982 | 0.982 |
|  | South China | **0.52 (0.38–0.71)** | **<0.001** | **<0.001** |
|  | Southwest China | 0.90 (0.66–1.22) | 0.493 | 0.634 |
|  | Northwest China | 0.93 (0.66–1.31) | 0.673 | 0.713 |
| **ICU beds** | ≤10 | Ref |  |  |
|  | 11–15 | 1.07 (0.80–1.42) | 0.648 | 0.713 |
|  | 16–23 | 0.69 (0.50–0.95) | 0.023 | 0.076 |
|  | ≥24 | 0.65 (0.44–0.95) | 0.025 | 0.076 |
| **Annual ICU admissions** | ≤330 | Ref |  |  |
|  | 331–568 | 0.91 (0.69–1.21) | 0.531 | 0.637 |
|  | 569–956 | 0.78 (0.57–1.08) | 0.131 | 0.295 |
|  | ≥957 | 0.87 (0.60–1.26) | 0.450 | 0.623 |
| **Nurse-to-bed ratio** | <2.5 | Ref |  |  |
|  | 2.5 to <3.0 | **0.61 (0.44–0.86)** | **0.005** | **0.032** |
|  | ≥3.0 | 0.82 (0.51–1.31) | 0.401 | 0.602 |

The sensitivity analysis included 1,888 complete cases: 732 ICUs increased UO monitoring frequency during AKI and 1,156 did not.

Increased monitoring frequency was defined as more frequent UO monitoring during AKI than under routine practice. ICUs already performing routine hourly monitoring had no opportunity for further escalation and were classified as not escalating; therefore, this full-sample analysis was treated as a sensitivity analysis.

The model was mutually adjusted for all variables shown. Routine UO monitoring frequency was not included because routine hourly monitoring structurally precluded further escalation; the primary ceiling-effect analysis was restricted to ICUs without routine hourly monitoring (Model C in Table 4).

Complete-case analysis was used. FDR-adjusted q values were calculated using the Benjamini–Hochberg method; bold values indicate q<0.05. Physician and nurse headcounts were excluded because of collinearity; nursing intensity was represented by the nurse-to-bed ratio.

Abbreviations: AKI, acute kidney injury; aOR, adjusted odds ratio; FDR, false discovery rate; ICU, intensive care unit; UO, urine output.

**Supplementary Table S6. Spearman correlation matrix for ICU resource variables**

| **Variable** | **Non-missing, n** | **1** | **2** | **3** | **4** | **5** |
| --- | --- | --- | --- | --- | --- | --- |
| 1. ICU beds | 1,895 | 1.000 |  |  |  |  |
| 2. ICU physicians | 1,893 | 0.818 (<0.001) | 1.000 |  |  |  |
| 3. ICU nurses | 1,894 | 0.864 (<0.001) | 0.877 (<0.001) | 1.000 |  |  |
| 4. Annual ICU admissions | 1,889 | 0.733 (<0.001) | 0.698 (<0.001) | 0.759 (<0.001) | 1.000 |  |
| 5. Nurse-to-bed ratio | 1,894 | -0.027 (0.241) | 0.294 (<0.001) | 0.435 (<0.001) | 0.207 (<0.001) | 1.000 |

Cells show Spearman’s ρ with two-sided P values in parentheses; the lower triangle is presented. Pairwise complete observations were used (n=1,887–1,894).

ICU beds, physicians, and nurses were strongly correlated (ρ=0.818–0.877). Therefore, physician and nurse headcounts were excluded from the final multivariable models; ICU beds represented capacity, annual ICU admissions represented activity, and the nurse-to-bed ratio represented nursing intensity.

The maximum variance inflation factor across the final Models A–D ranged from 2.87 to 3.01, indicating no substantial residual multicollinearity.

Abbreviations: ICU, intensive care unit; ρ, Spearman rank correlation coefficient.

**Supplementary Table S7. Components, scoring rules, and rationale for the exploratory AKI Monitoring Implementation Score (AKI-MIS)**

| **Domain** | **Component** | **Response option(s) and scoring** | **Raw score** | **Scoring rationale and interpretation** |
| --- | --- | --- | --- | --- |
| **1. Detection and surveillance structure** | AKI diagnosis implemented | Yes = 2; No = 0 | 0–2 | Captures the foundational ICU-level capability to recognize AKI. |
|  | AKI staging explicitly implemented | Yes = 2; No = 0 | 0–2 | Explicit staging supports severity classification and risk stratification. |
|  | Diagnostic basis | UO + SCr (50% each) = 2; mainly UO or mainly SCr = 1; missing or not classifiable = 0 | 0–2 | Use of both KDIGO diagnostic criteria receives full credit; reliance mainly on one criterion receives partial credit. |
|  | AKI high-risk early warning system | Yes = 2; No = 0 | 0–2 | Captures prospective risk-identification infrastructure; it is an implementation-capacity indicator, not a patient-outcome measure. |
|  | Routine eGFR calculation | Yes = 2; No = 0 | 0–2 | Captures routine renal-function assessment. eGFR does not substitute for UO or SCr criteria for AKI diagnosis. |
|  | **Domain 1 raw total** | **Sum item points; domain score = raw total / 10 × 25** | **0–10** | **The transformed score contributes 0–25 points to the total AKI-MIS.** |
| **2. Monitoring intensity and escalation** | Routine UO monitoring frequency | Every 1 h = 3; every 2–3 h = 2; every 4–8 h = 1; every 12/24 h or irregular = 0 | 0–3 | Greater frequency represents greater temporal resolution of routine surveillance; it is not interpreted as care quality by itself. |
|  | UO monitoring frequency in sepsis | Same scoring rule as for routine UO monitoring frequency | 0–3 | Captures risk-responsive surveillance in a common high-risk state. |
|  | UO monitoring frequency in AKI | Same scoring rule as for routine UO monitoring frequency | 0–3 | Captures surveillance after AKI recognition. Escalation from routine practice is analyzed separately to address the ceiling effect. |
|  | UO monitoring frequency in SA-AKI | Same scoring rule as for routine UO monitoring frequency | 0–3 | Captures surveillance in the combined high-risk state of sepsis-associated AKI. |
|  | Routine SCr monitoring frequency | <12 h or every 12 h = 3; daily = 2; every 2 days = 1; ≥3 days or irregular = 0 | 0–3 | Shorter intervals represent greater intensity of routine biochemical surveillance. |
|  | Higher-frequency SCr monitoring in high-risk states | Use the highest-frequency category across sepsis, AKI, and SA-AKI; apply the same SCr scoring rule as above | 0–3 | Summarizes whether enhanced biochemical surveillance is implemented in at least one high-risk state. |
|  | **Domain 2 raw total** | **Sum item points; domain score = raw total / 18 × 25** | **0–18** | **The transformed score contributes 0–25 points to the total AKI-MIS.** |
| **3. Standardization and guideline concordance** | UO frequency adjusted by illness severity | Yes = 2; No = 0 | 0–2 | Captures whether the monitoring protocol can adapt to clinical risk. |
|  | SCr frequency adjusted by illness severity | Yes = 2; No = 0 | 0–2 | Captures whether biochemical surveillance can adapt to clinical risk. |
|  | UO documentation unit | mL/kg/h = 3; mL/h = 2; other unit or mL = 0 | 0–3 | mL/kg/h directly operationalizes the weight-standardized KDIGO oliguria definition and therefore receives full guideline-concordance credit. mL/h remains clinically useful and receives partial credit; this hierarchy does not imply universal clinical superiority of one unit. |
|  | Oliguria threshold standardization | KDIGO-consistent threshold (<0.5 mL/kg/h for 6 h) = 3; other explicit mL/kg/h or mL/h threshold = 2; free-text/other threshold = 1; not classifiable = 0 | 0–3 | Full credit denotes concordance with the prespecified KDIGO reference; explicit alternative thresholds receive partial credit. This measures guideline alignment and does not assert that the KDIGO threshold is optimal for every patient. |
|  | **Domain 3 raw total** | **Sum item points; domain score = raw total / 10 × 25** | **0–10** | **The transformed score contributes 0–25 points to the total AKI-MIS.** |

**Supplementary Table S7. Components, scoring rules, and rationale for the exploratory AKI Monitoring Implementation Score (AKI-MIS) (continued)**

| **Domain** | **Component** | **Response option(s) and scoring** | **Raw score** | **Scoring rationale and interpretation** |
| --- | --- | --- | --- | --- |
| **4. Digitalization and response loop** | Monitoring mode | Dynamic monitoring for all patients = 3; dynamic monitoring for high-risk patients/other selected groups = 2; manual monitoring only = 0 | 0–3 | The hierarchy reflects breadth of automated implementation. Targeted high-risk monitoring receives substantial partial credit; lower points do not imply poorer bedside care. |
|  | Oliguria alert to physicians | Yes = 2; No = 0 | 0–2 | Captures the presence of a communication step in the detection-to-response loop. |
|  | Abnormal UO notification timeliness (manual-monitoring branch) | Notify immediately = 2; notify during handover = 1; notify when workload allows/other = 0 | 0–2 | Captures timeliness of the reported manual notification workflow. |
|  | Dynamic monitoring data uploaded to EMR | Yes = 2; No = 0 | 0–2 | Captures digital integration and availability of monitoring data in the clinical record. |
|  | Physician response after abnormal UO detection | Immediate intervention = 2; wait until AKI diagnostic criteria are met = 0 | 0–2 | Full credit reflects reported prompt activation of the clinical assessment/response loop, not mandatory treatment of every transient oliguria episode; clinical reassessment may precede intervention. |
|  | **Domain 4 raw total** | **Sum item points; domain score = raw total / 11 × 25** | **0–11** | **The transformed score contributes 0–25 points to the total AKI-MIS.** |

AKI-MIS is an investigator-developed, exploratory formative composite intended to summarize reported ICU-level implementation of AKI monitoring processes. Higher scores indicate broader implementation of the included processes; they do not directly measure patient outcomes, overall care quality, or the appropriateness of an individual clinical decision.

Each domain raw total is linearly transformed to 0–25 points (domain score = observed raw total / domain maximum × 25), and the four transformed domain scores are summed to yield a total score from 0 to 100. Equal domain contribution was prespecified to prevent domains containing more items from dominating the total numerically; this is a transparent design choice rather than an empirically derived claim that the four domains are clinically equivalent.

A sensitivity analysis using an alternative non-domain-equalized weighting is reported in Supplementary Table S8. In that analysis, all raw item points are summed (range, 0–49) and rescaled to 0–100, so domain contributions are proportional to their raw maxima (10, 18, 10, and 11 points, respectively).

The components are treated as formative indicators that jointly describe different aspects of implementation; they are not assumed to be interchangeable manifestations of a single latent trait. Consequently, internal-consistency statistics such as Cronbach’s alpha were not used to validate the score.

When an ICU reported using both dynamic and manual UO monitoring, the highest-scoring available response was used separately for the UO documentation-unit, oliguria-threshold, and physician-alert components. This prespecified rule avoided penalizing an ICU for retaining a parallel manual workflow.

The revised analyses use AKI-MIS only as a continuous exploratory measure; no categorical labels or cut points are applied. The score is not intended for ICU ranking, performance assessment, or bedside decision-making and requires external and prospective validation.

Abbreviations: AKI, acute kidney injury; AKI-MIS, AKI Monitoring Implementation Score; eGFR, estimated glomerular filtration rate; EMR, electronic medical record; ICU, intensive care unit; KDIGO, Kidney Disease: Improving Global Outcomes; SA-AKI, sepsis-associated AKI; SCr, serum creatinine; UO, urine output.

**Supplementary Table S8. Sensitivity analysis of AKI-MIS weighting: equal-domain versus non-domain-equalized raw-point scoring**

**Panel A. Score definition and distribution**

| **Measure** | **Primary equal-domain weighting** | **Alternative raw-point weighting** |
| --- | --- | --- |
| **Scoring formula** | Σ[(domain raw score/domain maximum) × 25] | (Σ raw item points/49) × 100 |
| **Maximum contribution of Domains 1/2/3/4** | 25.0% / 25.0% / 25.0% / 25.0% | 20.4% / 36.7% / 20.4% / 22.4% |
| **ICUs, n** | 1,896 | 1,896 |
| **Mean (SD)** | 67.07 (11.42) | 68.02 (12.07) |
| **Median (IQR)** | 68.08 (60.30–74.91) | 69.39 (61.22–75.51) |
| **Range** | 19.09–100.00 | 16.33–100.00 |

**Panel B. Individual-level agreement between weighting approaches**

| **Agreement measure** | **Result** |
| --- | --- |
| Pearson correlation coefficient | 0.980 |
| Spearman rank correlation coefficient | 0.981 |
| Lin concordance correlation coefficient | 0.975 |
| Mean paired difference, alternative minus primary (95% CI), points | 0.95 (0.84–1.06) |
| Mean absolute difference, points | 2.14 |
| Median absolute difference, points | 1.89 |
| 95th percentile of absolute difference, points | 4.91 |
| Maximum absolute difference, points | 9.05 |
| Scores within 2.5 points, % | 64.5 |
| Scores within 5 points, % | 95.6 |
| Scores within 10 points, % | 100.0 |

**Supplementary Table S8. Sensitivity analysis of AKI-MIS weighting:
equal-domain versus non-domain-equalized raw-point scoring (continued)**

**Panel C. Multivariable determinants under the two weighting approaches**

| **Characteristic** | **Level** | **Primary adjusted difference (95% CI)** | **FDR q** | **Alternative adjusted difference (95% CI)** | **FDR q** |
| --- | --- | --- | --- | --- | --- |
| **Hospital level** | Tertiary | Ref |  | Ref |  |
|  | Non-tertiary | -0.63 (-2.12–0.85) | 0.661 | -0.86 (-2.47–0.74) | 0.583 |
| **Teaching hospital** | No | Ref |  | Ref |  |
|  | Yes | **2.36 (1.16–3.56)** | **0.002** | **2.33 (1.05–3.61)** | **0.007** |
| **Hospital type** | General | Ref |  | Ref |  |
|  | Specialty | -0.10 (-2.26–2.07) | 0.973 | -0.05 (-2.31–2.21) | 0.965 |
| **ICU type** | General ICU | Ref |  | Ref |  |
|  | Specialised ICU | -0.40 (-2.17–1.36) | 0.843 | -0.74 (-2.62–1.14) | 0.621 |
| **Region** | East China | Ref |  | Ref |  |
|  | North China | 0.05 (-1.55–1.66) | 0.973 | 0.51 (-1.21–2.23) | 0.686 |
|  | Northeast China | 0.03 (-1.75–1.81) | 0.973 | 0.55 (-1.35–2.45) | 0.686 |
|  | Central China | 0.77 (-1.77–3.31) | 0.762 | 0.55 (-2.06–3.15) | 0.757 |
|  | South China | -0.52 (-2.14–1.10) | 0.762 | 0.64 (-1.02–2.30) | 0.621 |
|  | Southwest China | 0.11 (-1.59–1.82) | 0.973 | 0.34 (-1.47–2.14) | 0.757 |
|  | Northwest China | 1.09 (-0.72–2.90) | 0.493 | 0.96 (-0.96–2.88) | 0.589 |
| **ICU beds** | ≤10 | Ref |  | Ref |  |
|  | 11–15 | 0.69 (-0.94–2.33) | 0.661 | 0.74 (-1.00–2.49) | 0.621 |
|  | 16–23 | 1.79 (0.06–3.51) | 0.252 | 1.79 (-0.07–3.64) | 0.265 |
|  | ≥24 | 2.68 (0.63–4.73) | 0.093 | 2.75 (0.58–4.92) | 0.118 |
| **Annual ICU admissions** | ≤330 | Ref |  | Ref |  |
|  | 331–568 | 1.09 (-0.46–2.64) | 0.493 | 1.18 (-0.48–2.85) | 0.422 |
|  | 569–956 | 1.30 (-0.47–3.07) | 0.493 | 1.65 (-0.24–3.53) | 0.313 |
|  | ≥957 | 1.22 (-0.77–3.22) | 0.493 | 1.69 (-0.42–3.79) | 0.352 |
| **Nurse-to-bed ratio** | <2.5 | Ref |  | Ref |  |
|  | 2.5 to <3.0 | 0.93 (-0.64–2.51) | 0.493 | 1.55 (-0.06–3.16) | 0.265 |
|  | ≥3.0 | 1.60 (-0.81–4.02) | 0.493 | 1.66 (-0.82–4.14) | 0.428 |

The primary AKI-MIS assigns each of the four domains an equal maximum contribution of 25 points. The alternative score sums all prespecified raw item points (range, 0–49) before rescaling to 0–100; therefore, domain contributions are proportional to their raw maxima. Item definitions and within-item point assignments were unchanged.

Panel C used 1,888 complete cases for both models. Multivariable ordinary least-squares regression with HC3 robust standard errors was mutually adjusted for all characteristics shown. FDR-adjusted q values were calculated separately within each model using the Benjamini–Hochberg method; bold values indicate q<0.05.

Adjusted R² was 0.030 for the primary score and 0.034 for the alternative score. Coefficient direction was concordant for 17 of 18 contrasts (94.4%). The only sign change was for South China versus East China, which was not significant under either weighting. Teaching-hospital status remained FDR-significant under both approaches.

Physician and nurse headcounts were excluded because of collinearity; ICU beds represented capacity, annual ICU admissions represented activity, and the nurse-to-bed ratio represented nursing intensity. No categorical AKI-MIS cut points were used.

Abbreviations: AKI, acute kidney injury; AKI-MIS, AKI Monitoring Implementation Score; CI, confidence interval; FDR, false discovery rate; ICU, intensive care unit; IQR, interquartile range; SD, standard deviation.

**Supplementary Table S9. Reported use, combinations, and regional variation in AKI biomarkers (N=1,896)**

**Panel A. Individual biomarker use and co-use patterns**

| **Section** | **Biomarker or combination pattern** | **ICU units, n (%)** |
| --- | --- | --- |
| **Individual markers** | NGAL | 330 (17.4) |
|  | KIM-1 | 143 (7.5) |
|  | IL-18 | 281 (14.8) |
|  | Cystatin C | 1,334 (70.4) |
|  | [TIMP-2]·[IGFBP7] | 72 (3.8) |
|  | L-FABP | 42 (2.2) |
|  | Other | 450 (23.7) |
| **Number of listed biomarkers** | 0 prespecified named biomarkers (“Other” selected) | 359 (18.9) |
|  | 1 listed biomarker | 1,116 (58.9) |
|  | 2 listed biomarkers | 292 (15.4) |
|  | 3 listed biomarkers | 66 (3.5) |
|  | ≥4 listed biomarkers | 63 (3.3) |
| **Most frequent patterns** | Cystatin C as the only prespecified named biomarker | 954 (50.3) |
|  | No prespecified named biomarker (“Other” selected) | 359 (18.9) |
|  | NGAL + Cystatin C | 128 (6.8) |
|  | IL-18 + Cystatin C | 111 (5.9) |
|  | NGAL only | 87 (4.6) |
|  | IL-18 only | 58 (3.1) |
|  | All other named-marker patterns | 199 (10.5) |

**Supplementary Table S9. Reported use, combinations, and regional variation in AKI biomarkers
(N=1,896; continued)**

**Panel B. Regional variation in reported biomarker use**

| **Region** | **NGAL** | **KIM-1** | **IL-18** | **Cystatin C** | **[TIMP-2]· [IGFBP7]** | **L-FABP** | **Other** |
| --- | --- | --- | --- | --- | --- | --- | --- |
| North China (n=242) | 48 (19.8) | 12 (5.0) | 28 (11.6) | 152 (62.8) | 8 (3.3) | 5 (2.1) | 76 (31.4) |
| Northeast China (n=198) | 29 (14.6) | 18 (9.1) | 22 (11.1) | 132 (66.7) | 7 (3.5) | 4 (2.0) | 54 (27.3) |
| East China (n=655) | 146 (22.3) | 56 (8.5) | 108 (16.5) | 463 (70.7) | 31 (4.7) | 15 (2.3) | 142 (21.7) |
| Central China (n=103) | 32 (31.1) | 9 (8.7) | 19 (18.4) | 80 (77.7) | 4 (3.9) | 3 (2.9) | 15 (14.6) |
| South China (n=283) | 26 (9.2) | 12 (4.2) | 30 (10.6) | 203 (71.7) | 4 (1.4) | 3 (1.1) | 70 (24.7) |
| Southwest China (n=241) | 25 (10.4) | 18 (7.5) | 42 (17.4) | 183 (75.9) | 9 (3.7) | 7 (2.9) | 45 (18.7) |
| Northwest China (n=174) | 24 (13.8) | 18 (10.3) | 32 (18.4) | 121 (69.5) | 9 (5.2) | 5 (2.9) | 48 (27.6) |
| **Global P** | <0.001 | 0.103 | 0.033 | 0.025 | 0.312 | 0.824 | 0.002 |
| **FDR q** | **<0.001** | 0.145 | 0.057 | 0.057 | 0.363 | 0.824 | **0.008** |

Data are n (%) of ICU units. Respondents could select multiple biomarkers; therefore, percentages for individual markers sum to more than 100%. Co-use patterns were defined using the six prespecified named biomarkers and excluded the “Other” option. “Other” could be selected with or without a named biomarker. Although the questionnaire displayed an “Other” write-in field, the exported analytic dataset did not retain a separately analysable text variable; therefore, these responses could not be further classified.

Regional differences were assessed separately for each marker using Pearson's chi-square test. For [TIMP-2]·[IGFBP7] and L-FABP, Monte Carlo P values based on 1,000,000 fixed-margin replicates were used because of sparse expected cell counts. FDR-adjusted q values were calculated across the seven biomarker-specific regional tests using the Benjamini-Hochberg method; bold values indicate q<0.05.

The survey recorded reported biomarker use but did not collect clinical indications, testing frequency, assay platform, decision thresholds, costs, or reimbursement information. Accordingly, regional variation should not be interpreted as evidence of cost-related differences.

Abbreviations: AKI, acute kidney injury; FDR, false discovery rate; ICU, intensive care unit; IGFBP7, insulin-like growth factor-binding protein 7; IL-18, interleukin 18; KIM-1, kidney injury molecule 1; L-FABP, liver-type fatty acid-binding protein; NGAL, neutrophil gelatinase-associated lipocalin; TIMP-2, tissue inhibitor of metalloproteinases 2.

**Supplementary Figure S1. Flow of survey responses, data-quality assessment, duplicate screening, and derivation of the final analytic sample.**

Ten records with resolvable logical inconsistencies were recoded and retained. After manual adjudication, 106 duplicate submissions were removed, leaving 1,896 unique adult ICU units for analysis. ICU, intensive care unit.
